# The American Electro-Therapeutic Association

**Published:** 1891-03

**Authors:** 


### Society Notes and Meetings.

The American Electro-Therapeutic Association was organized on the 22d of January, 1891, at the Academy of Medicine, No. 17 West 43d street, New York, by the adoption of a constitution and by-laws, and the election of the following officers: President, G. Betton Massey, M. D., Philadelphia; Vice-Presidents, William James Morton, M. D., and Augustin H. Goelet, M. D., New York; Secretary, William H. Walling, M. D., Philadelphia; Treasurer, George H. Rohé, M. D., Baltimore. Executive Council: Horatio R. Bigelow, M. D., Philadelphia; Franklin H. Martin, M. D., Chicago; William F. Hutchinson, M. D., Providence, R. I.; Frederic Peterson, M. D., New York, and Chauncey D. Palmer, M. D., Cincinnati, O.

